# Immunothrombosis and COVID-19 ‒ a nested post-hoc analysis from a 3186 patient cohort in a Latin American public reference hospital

**DOI:** 10.1016/j.clinsp.2023.100178

**Published:** 2023-02-28

**Authors:** Clarice Antunes de Lima, Fabio Augusto Rodrigues Gonçalves, Bruno Adler Maccagnan Pinheiro Besen, Antônio José Rodrigues Pereira, Sandro Félix Perazzio, Evelinda Marramon Trindade, Luiz Augusto Marcondes Fonseca, Nairo Massakazu Sumita, Vanusa Barbosa Pinto, Alberto José da Silva Duarte, Carolina Broco Manin, Arnaldo Lichtenstein

**Affiliations:** a Divisao de Farmacia, Instituto Central, Hospital das Clinicas HCFMUSP, Faculdade de Medicina, Universidade de Sao Paulo, Sao Paulo, SP, Brazil; b Laboratorio de Cirurgia Cardiovascular e Fisiopatologia da Circulacao (LIM11), Faculdade de Medicina FMUSP, Universidade de Sao Paulo, SP, Brazil; c Unidade de Terapia Intensiva, Disciplina de Emergencias Clinicas, Faculdade de Medicina FMUSP, Universidade de Sao Paulo, Sao Paulo, SP, Brazil; d Superintendencia, Hospital das Clinicas HCFMUSP, Faculdade de Medicina, Universidade de Sao Paulo, Sao Paulo, SP, Brazil; e Divisao de Laboratorio Central, Hospital das Clinicas HCFMUSP, Faculdade de Medicina, Universidade de Sao Paulo, Sao Paulo, SP, Brazil; f Nucleo de Avaliacao de Tecnologia em Saude, Hospital das Clinicas HCFMUSP, Faculdade de Medicina, Universidade de Sao Paulo, Sao Paulo, SP, Brazil; g Servico de Imunologia Clinica e Alergia, Hospital das Clinicas HCFMUSP, Faculdade de Medicina, Universidade de Sao Paulo, Sao Paulo, SP, Brazil; h Laboratorio de Dermatologia e Imunodeficiencias (LIM56), Faculdade de Medicina FMUSP, Universidade de Sao Paulo, Sao Paulo, SP, Brazil; i Departamento de Medicina Interna, Instituto Central, Hospital das Clinicas HCFMUSP, Faculdade de Medicina, Universidade de Sao Paulo, Sao Paulo, SP, Brazil

**Keywords:** COVID-19, Biomarkers, Cohort studies, Venous thromboembolism, Immunothrombosis

## Abstract

**Objective:**

COVID-19 is associated with an elevated risk of thromboembolism and excess mortality. Difficulties with best anticoagulation practices and their implementation motivated the current analysis of COVID-19 patients who developed Venous Thromboembolism (VTE).

**Method:**

This is a post-hoc analysis of a COVID-19 cohort, described in an economic study already published. The authors analyzed a subset of patients with confirmed VTE. We described the characteristics of the cohort, such as demographics, clinical status, and laboratory results. We tested differences amid two subgroups of patients, those with VTE or not, with the competitive risk Fine and Gray model.

**Results:**

Out of 3186 adult patients with COVID-19, 245 (7.7%) were diagnosed with VTE, 174 (5.4%) of them during admission to the hospital. Four (2.3% of these 174) did not receive prophylactic anticoagulation and 19 (11%) discontinued anticoagulation for at least 3 days, resulting in 170 analyzed. During the first week of hospitalization, the laboratory most altered results were C-reactive protein and D-dimer. Patients with VTE were more critical, had a higher mortality rate, worse SOFA score, and, on average, 50% longer hospital stay.

**Conclusion:**

Proven VTE incidence in this severe COVID-19 cohort was 7.7%, despite 87% of them complying completely with VTE prophylaxis. The clinician must be aware of the diagnosis of VTE in COVID-19, even in patients receiving proper prophylaxis.

## Highlights

• VTE COVID-19 patients were sicker, their hospital stay was longer, and they were more likely to die.
• VTE incidence is still considerable in COVID-19 patients even under pharmacological prophylaxis.
• Prophylaxis and treatment compliance are essential to achieve a low VTE incidence.

## Introduction

COVID-19 is a multisystem inflammatory disease leading to high mortality rates in the early phase of the pandemic, due to the aggressiveness of its strains and the absence of developed and tested vaccines. Several studies have associated COVID-19 with a high incidence of thrombotic phenomena^1^^,^^2^ and excess mortality.^3^^,^^4^ The elevated risk of thromboembolism has been attributed^5^ to hyper-inflammatory states and cytokine storm, resulting in induction of sepsis-related coagulopathies, disseminated intravascular coagulation, platelet dysfunction,^6^ endothelitis, and local thrombosis or micro thrombosis.^7^ Some studies have shown that prophylaxis with full doses of anticoagulant agents in moderate to severely ill patients improved survival rates.^8^ However, this outcome was not confirmed in critically ill patients hospitalized in intensive care units with ventilatory support.^8^^,^^9^ In contrast, in moderately ill patients, therapeutic-dose anticoagulants were found to provide a survival benefit in the prevention and treatment of Venous Thromboembolism (VTE) until hospital discharge, but more major bleeding occurred than with conventional doses of thromboprophylaxis (1.9% vs. 0.9%).^10^

The association of elevated risk of thromboembolism in patients with viral or septic inflammatory disease had already been described years before the COVID-19 pandemic.^11^, ^12^, ^13^ In critical and noncritical patients, the mechanisms potentially involved in this association, named immunothrombosis,^14^ have gradually been clarified.^15^^,^^16^

During the COVID-19 pandemic, however, the application of this knowledge became difficult for those involved in direct patient care due to the multiplicity and variation in the recommendations for thromboprophylaxis,^17^^,^^18^ as well as the low or very low quality of the studies that such recommendations were based on.^19^^,^^20^ One reason may be due to the COVID-19 infection itself and its severe disease pathogenesis, which was then (and still is) in the discovery phase. In addition, the risk stratification approaches used among the patients studied and the anticoagulation strategies, with their recommended drugs and dosages, showed significant heterogeneity. Despite the high volume of publications on this topic to this date, there is an expressed requirement^17^ for greater and better knowledge about these patients who develop Venous Thromboembolism (VTE) and about the VTE impact in their clinical evolution, aiming to improve patient safety and subsidize interventions with less undesirable effects.

The present study aimed to perform an analysis of patients diagnosed with VTE within a cohort of patients admitted to a tertiary, public, and reference hospital in Latin America that was completely dedicated to the care of patients with COVID-19 in the period analyzed.

## Methods

### Study design

This is a post-hoc analysis of a cohort economic study,^21^ undertaken at Instituto Central, Hospital das Clinicas HCFMUSP, Faculdade de Medicina, Universidade de Sao Paulo, Sao Paulo, SP, Brazil. The HCFMUSP is a tertiary, university-based medical facility. During the study period, only patients with moderate and severe COVID-19 cases, referenced by the Regulation System of the Sao Paulo State Health Secretariat, were admitted to the HCFMUSP, which had 900 ward beds and 300 intensive care beds for this purpose. The HCFMUSP COVID-19 cohort was constituted to facilitate multidisciplinary studies addressing medical, functional, and neuropsychiatric outcomes. The study protocol follows the Declaration of Helsinki, was approved by the HCFMUSP Ethics Committee (CAPPesq-HC #4.107.580), and is reported according to The Strengthening the Reporting of Observational Studies in Epidemiology (STROBE) Statement.^22^

The authors included adult (age ≥18 years) patients with a confirmed diagnosis of COVID-19, admitted from March 30, 2020, to June 30, 2020. Confirmed COVID-19 was defined as a positive real-time Reverse-Transcriptase Polymerase Chain Reaction (RT-PCR) from a nasal and/or throat swab together with pathognomonic signs, symptoms, or radiological findings of COVID-19 pneumonia, as previously described.^21^

The established institutional prophylactic anticoagulation protocol recommended using weight-adjusted low-dose Low Molecular Weight derivative (LMW) (enoxaparin), and the use of unfractionated heparin if LMW was contraindicated or for patients with renal dysfunction. The scarce mechanical compressive equipment was reserved for critical patients with an anticoagulation absolute contraindication.

The strategy the authors employed to find patients with thrombosis was to look for those who received full doses of anticoagulants since the electronic linking between the radiology service and the electronic patients’ records was operational. In this way, the authors did not need to review all patients’ records, just those belonging to patients treated with full doses of anticoagulants, either enoxaparin or unfractionated heparin. After this first screening, the authors reviewed the clinical records of those meeting the inclusion criteria. Patients with confirmed VTE by chest angiotomography and/or venous ultrasound were selected and further stratified as Pulmonary Embolism (PE) and Deep Vein Thrombosis (DVT), respectively. Patients who received full doses of anticoagulants but did not develop venous thromboembolism were not included in the study, because they could previously be on anticoagulants for other reasons.

Patients’ demographic data, as well as their disease severity, categorized by the worst modified Sequential Organ Failure Assessment Score (mSOFA), the SOFA score^23^ minus the neurologic component, observed during hospitalization, length of hospital stay, need for kidney replacement therapy and incidence of mortality. Information about previous treatments was not available. Laboratory parameters, such as fibrinogen concentration and platelets number were categorized according to the maximum point of the area under the intersection between sensitivity and specificity in the Receiver Operator Characteristic curve (ROC curve). A D-dimer 4,000 ng/mL threshold and a CRP 220 mg/dL threshold were adopted, as they were previously associated with the highest mortality.^24^ Two groups of COVID-19 patients were compared: G1 with confirmed VTE versus G2 without VTE during hospitalization.

### Statistical analysis

In the present study, no sample size calculation was performed, since the authors used a convenience sample nested in the described cohort, according to the inclusion criteria.

In the descriptive analysis, continuous variables are presented as means and Confidence Intervals (95% CI). Categorical variables are summarized as absolute or relative distributions, cases counted, or proportions related to their relevant denominator, respectively. The time when events occurred during the hospital stay is presented in time series analysis box-scatter plots. The subgroups were compared, and the significance of the differences observed was tested employing the Fisher's Exact Test or Chi-Square test and Wilcoxon Rank Sum Test for continuous and categorical variables, for counted data and independent samples.

To evaluate the risk of VTE accounting for the competing risk of death, the authors used the Fine and Gray model and present sub-distribution hazard ratios and the cumulative incidence of VTE accounting for death. In this model, we included demographic variables (age, sex, color/ ethnicity) and clinical variables, including mSOFA, acute kidney injury occurrence, and chronic kidney disease. We then added the worst CRP and D-dimer levels in the first 5 days of hospitalization in this same model to evaluate the association of these analytes with the incidence of VTE. We selected variables a priori based on clinical reasoning.

All statistical tests were two-sided. Missing data were not imputed. Statistical analyses were performed using R (v4.1.0).^25^ A p-value < 0.05 was considered statistically significant.

## Results

**Fig. 1:**
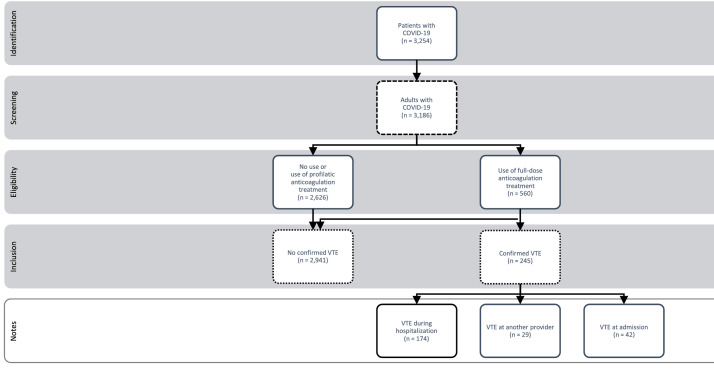
Flowchart of study participants. Note: Venous Thromboembolism (VTE).

From March 30 to June 30, 2020, 3,254 patients were admitted with confirmed COVID-19. More than half of this cohort, approximately 52%, required intensive care as described in a previous publication.^21^ Amid them, there were 3,186 adult patients. The flow of selection of the subgroups of patients studied is outlined in Fig. 1.

There were 560 patients who escalated anticoagulant doses. Among them, there were 245 patients (44%) who had VTE confirmed, 42 (17%) had VTE diagnosed at hospital admission, 29 (12%) were transferred to the hospital having a VTE diagnosis established, and 174 (71%) had confirmed VTE during hospitalization in the authors’ service. These 245 patients were compared to the 2,941 patients who did not have VTE (Table 1).

**Table 1:** Patients’ characteristics stratified by the occurrence of Venous Thromboembolism (VTE).

Characteristics	VTE No (n = 2,941)	VTE Yes (n = 245)	Total (n = 3,186)	p-value
Sex (male)	1,601 (54.4%)	146 (59.6%)	1,747 (54.8%)	0.1
Age (years)				0.2
18 (40)	456 (15.5%)	31 (12.7%)	487 (15.3%)	
40 (55)	736 (25.0%)	56 (22.9%)	792 (24.9%)	
55 (101)	1,749 (59.5%)	158 (64.5%)	1,907 (59.9%)	
Race/Colour				0.4
White	1,885 (64.1%)	143 (58.4%)	2,028 (63.7%)	
Black	214 (7.3%)	21 (8.6%)	235 (7.4%)	
Brown	700 (23.8%)	68 (27.8%)	768 (24.1%)	
Yellow	26 (0.9%)	2 (0.8%)	28 (0.9%)	
Unknown	116 (3.9%)	11 (4.5%)	127 (4.0%)	
Weight (kg)				< 0.01
Median (Q1, Q3)	72 (64, 77)	69 (61, 77)	72 (64, 77)	
Mean (CI)	74 (73, 74)	70 (69, 72)	73 (73, 74)	
Length of stay (days)				< 0.01
Median (Q1, Q3)	10 (6, 18)	22 (13, 34)	11 (6, 19)	
Mean (CI)	14 (13, 14)	27 (25, 29)	15 (14, 15)	
Acute kidney injury (yes)	378 (12.9%)	53 (21.6%)	431 (13.5%)	< 0.01
Chronic kidney failure (yes)	212 (7.2%)	15 (6.1%)	227 (7.1%)	0.6
Mortality (yes)	845 (28.7%)	90 (36.7%)	935 (29.3%)	< 0.01
Worst mSOFA during hospitalization (score)				< 0.01
Median (Q1, Q3)	3 (0, 8)	8 (3, 11)	4 (1, 9)	
Mean (CI)	5 (5, 5)	7 (6, 8)	5 (5, 5)	
Worst mSOFA in the first five days (score)				
Median (Q1, Q3)	3 (0, 8)	5 (3, 8)	3 (1, 8)	
Mean (CI)	4 (4, 4)	6 (5, 6)	4 (4, 4)	
D-dimer (ng/mL FEU)				< 0.01
Median (Q1, Q3)	2,044 (1,015, 6,403)	7,436 (3,336, 33,842)	2,219 (1,054, 6,954)	
Mean (CI)	8,723 (7,966, 9,479)	25,635 (21,014, 30,257)	10,074 (9,267, 10,881)	
Altered D-dimer (≥ 4,000 ng/mL) (yes)	812 (35.6%)	138 (69.7%)	950 (38.3%)	< 0.01
C-reactive protein (mg/dL)				< 0.01
Median (Q1, Q3)	167 (74, 293)	298 (145, 356)	174 (79, 304)	
Mean (CI)	192 (186, 197)	273 (253, 292)	198 (192, 203)	
Altered C-reactive protein (≥ 220 mg/dL) (yes)	952 (38.0%)	134 (64.1%)	1086 (40.1%)	< 0.01

Q1, 1^st^ Quantile (25%); Q3, 3^rd^ Quantile (75%); CI, Confidence Interval.

Patients who developed VTE tended to be older and thinner and had significant indices of more severe clinical condition, expressed by worse scores on the maximum mSOFA scale and greater occurrence of acute kidney injury, leading to a significantly prolonged hospital stay (average of 27 days) and excess mortality greater than 8%. However, in a multivariate analysis with death as an outcome, the variables standing out as significant were the severity score SOFA and laboratory data, such as a C-reactive protein over 220 mg/dL and a D-dimer over 4000 (Tables 2 and 3).

**Table 2:** Risk factors for VTE accounting for the competing risk of death by the Fine-Gray model.

Characteristic	SHR	95% CI	p-value
Male Sex	1.13	0.97, 1.46	0.4
Age ≥ 70 years	1.10	0.84, 1.45	0.5
Race/Colour			0.9
White	‒	‒	
Black	1.23	0.94, 1.61	0.1
Other	1.28	0.73, 2.25	0.4
Acute kidney injury	0.93	0.64, 1.35	0.7
Chronic kidney disease	0.60	0.35, 1.05	0.07
Worst mSOFA in the first five days (score)	1.05	1.01, 1.09	< 0.01

**Table 3:** Risk factors for VTE accounting for the competing risk of death by the Fine-Gray model with altered D-dimer and C-reactive protein.

Characteristic	SHR	95% CI	p-value
Male Sex	1.08	0.97, 1.47	0.6
Age ≥ 70 years	1.27	0.93, 1.74	0.1
Race/Colour			
White	‒	‒	
Black	1.10	0.80, 1.53	0.6
Other	1.27	0.68, 2.37	0.5
Acute kidney injury (yes)	0.95	0.61, 1.47	0.8
Chronic kidney disease (yes)	0.71	0.37, 1.38	0.3
Worst mSOFA in the first five days (score)	0.97	0.93, 1.02	0.2
Altered D-dimer in the first five days (≥ 4,000 ng/mL) (yes)	2.36	1.65, 3.36	< 0.01
Altered C-reactive protein in the first five days (≥ 220 mg/dL) (yes)	1.79	1.26, 2.54	< 0.01

SHR, Sub distribution Hazard Ratio; CI, Confidence Interval.

**Fig. 2:**
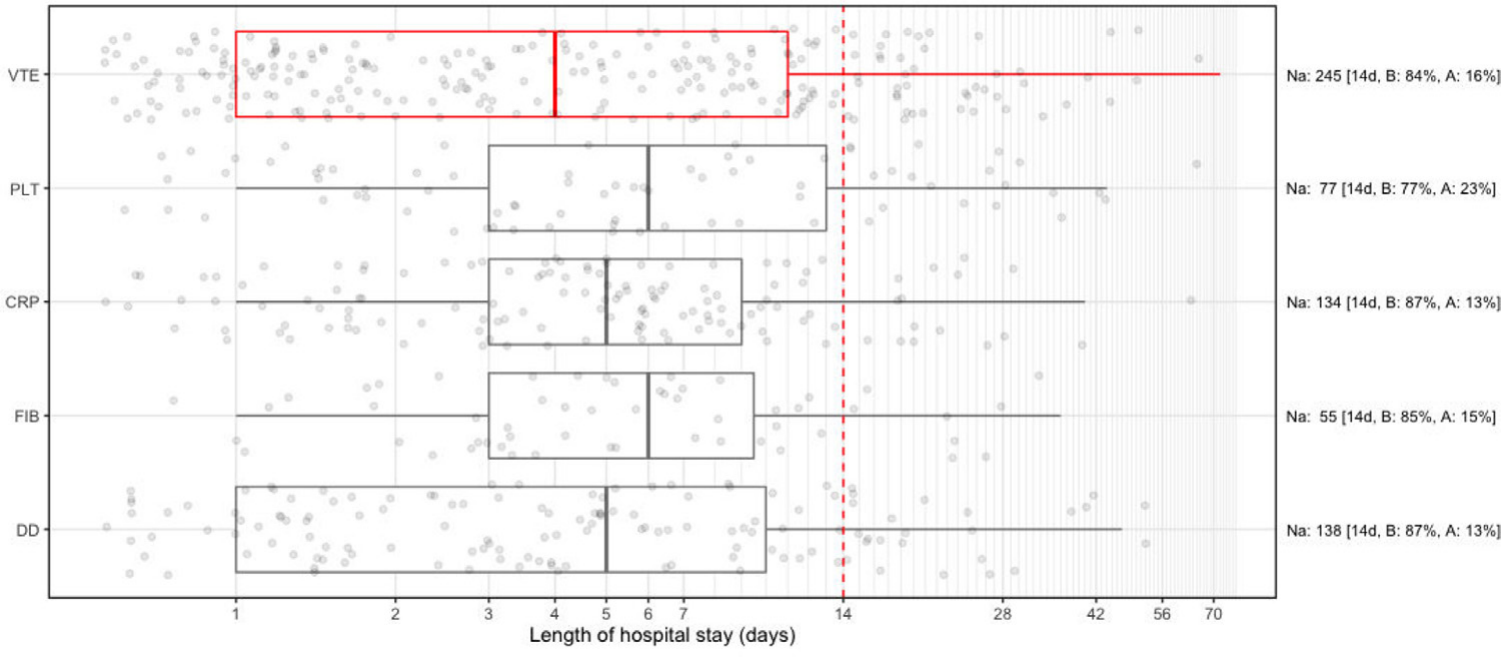
Time analysis of Venous Thromboembolism (VTE) diagnosis and of the highest (CRP, C-Reactive Protein; DD, D-Dimer; FIB, Fibrinogen) and lowest analytes serum levels (Platelet count, PLT) during hospitalization. Note: Na, Number of patients with VTE and altered test results; (B) Percentage of patients before 14 days; (A) Percentage of patients after 14 days.

The time series analysis of the lowest platelet count, D-dimer and fibrinogen serum levels is shown in Fig. 2. Those occurrences reflect the moment of hospitalization when the patients developed VTE. Most of those alterations occurred in the first week of hospitalization, regardless of whether the patient was in an intensive care unit or in a ward.

**Fig. 3:**
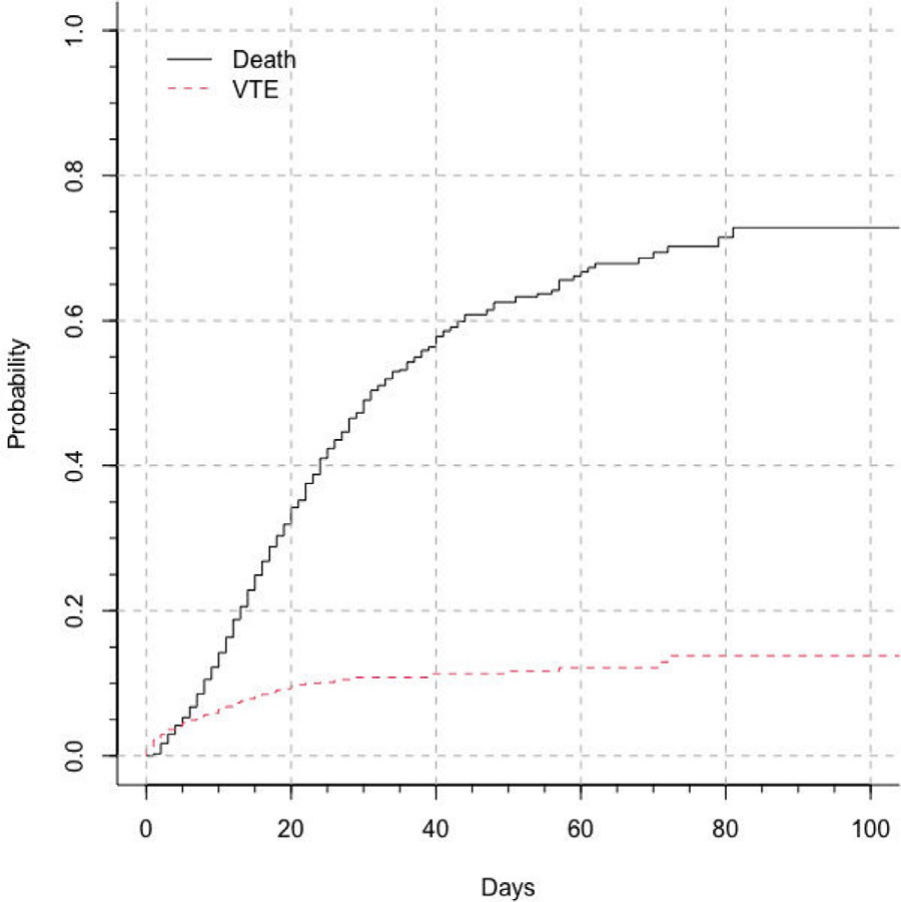
Cumulative incidence (risk over time) of Venous Thromboembolism (VTE) accounting for the risk of death during hospitalization.

During hospitalization in the center, 174 patients had a VTE event, among whom 128 received enoxaparin as prophylaxis (74%) and 46 received prophylactic unfractionated heparin (26%). In addition, amid them, VTE was diagnosed as Pulmonary Embolism (PE) in 98 (56%) and as Deep Vein Thrombosis (DVT) in 76 (44%). Among the total population of 245 patients with a confirmed VTE diagnosis, 144 had PE (59%) and 101 (41%) had DVT. The cumulative incidence of VTE accounting for the competing risk of death is presented in Fig. 3.

Venous thromboembolism prophylaxis was administered for 170 of 174 patients who had VTE inside the hospital, with an 11% occurrence of compliance discontinuity. Table 4 presents the characteristics of those 170 patients who developed VTE and missed three or more consecutive days of prophylaxis (19 patients), compared to patients who developed VTE and did not miss any dose or missed less than three consecutive days of prophylaxis (151 patients). This comparison demonstrates that there were no significant differences between these 2 subgroups regarding age, sex, weight, analyte changes, or clinical conditions. However, discontinuing 3 or more consecutive days of prophylaxis had a statistically significant impact on the length of hospital stay. Severe bleeding is one of the reasons for indicating the discontinuation of prophylaxis and, despite a greater bleeding proportion documented in the group who discontinued prophylaxis for 3 or more consecutive days, this difference was not statistically significant, perhaps due to its very low incidence among studied patients.

**Table 4:** Characteristics of patients who had venous thromboembolism at service and received prophylaxis at some point during hospital stay. Comparison of patients who missed less than 3 Consecutive Doses (< 3CDDL) with those who missed 3 or more consecutive doses (≥ 3 CDDL).

Characteristics	< 3 CDDL (n = 151)	≥ 3 CDDL (n = 19)	Total (n = 170)	p-value
Sex (male)	89 (58.9%)	13 (68.4%)	102 (60.0%)	0.4
Age (years)				0.3
[18, 40)	15 (9.9%)	4 (21.1%)	19 (11.2%)	
[40, 55]	38 (25.2%)	4 (21.1%)	42 (24.7%)	
(55, 101]	98 (64.9%)	11 (57.9%)	109 (64.1%)	
Race/Colour				0.9
White	89 (58.9%)	12 (63.2%)	101 (59.4%)	
Black	9 (6.0%)	1 (5.3%)	10 (5.9%)	
Brown	44 (29.1%)	6 (31.6%)	50 (29.4%)	
Yellow	0 (0.0%)	0 (0.0%)	0 (0.0%)	
Unknown	9 (6.0%)	0 (0.0%)	9 (5.3%)	
Weight (kg)				0.4
Median (Q1, Q3)	69 (62, 77)	66 (60, 74)	68 (62, 77)	
Mean (CI)	70 (69, 72)	70 (62, 77)	70 (69, 72)	
Length of stay (days)				< 0.001
Median (Q1, Q3)	24 (17, 35)	44 (30, 50)	25 (18, 40)	
Mean (CI)	29 (26, 33)	44 (35, 53)	31 (28, 34)	
Acute kidney injury (yes)	39 (25.8%)	6 (31.6%)	45 (26.5%)	0.5
Mortality (yes)	49 (32.5%)	7 (36.8%)	56 (32.9%	0.7
Severe bleeding (yes)	3 (2.0%)	1 (5.3%)	4 (2.4%)	0.3
Worst mSOFA during hospitalization (score)				0.2
Median (Q1, Q3)	8 (4, 11)	10 (8, 12)	8 (5, 11)	
Mean (CI)	8 (7, 8)	9 (7, 11)	8 (7, 8)	
Worst mSOFA in the first five days (score)				0.6
Median (Q1, Q3)	7 (4, 9)	7 (4, 10)	7 (4, 9)	
Mean (CI)	6 (6, 7)	7 (5, 9)	6 (6, 7)	
D-dimer (ng/mL FEU)				0.3
Median (Q1, Q3)	13,846 (4,024, 45,508)	7,086 (3,737, 20,021)	12,993 (3,990, 37,980)	
Mean (CI)	28,671 (22,708, 34,633)	19,942 (3,820, 36,065)	27,618 (22,074, 33,162)	
Altered D-dimer (≥4,000ng/mL) (yes)	93 (75.0%)	12 (70.6%)	105 (74.5%)	0.7
C-reactive protein (mg/dL)				0.8
Median (Q1, Q3)	316 (207, 374)	319 (188, 350)	316 (204, 374)	
Mean (CI)	302 (278, 326)	293 (228, 357)	301 (279, 323)	
Altered C-reactive protein (≥220 mg/dL) (yes)	91 (72.2%)	12 (70.6%)	103 (72.0%)	> 0.9

Q1, 1^st^ Quantile (25%); Q3, 3^rd^ Quantile (75%); CI, Confidence Interval; Msofa, modified SOFA Score.

Mortality of VTE patients was higher (36.7%) than that of non-VTE patients (28.75%) regardless of the place of occurrence, whether at our hospital or elsewhere (Table 1).

## Discussion

The VTE incidence was 7.7% (245/3,186) in this post-hoc analysis, with 5.3% (174/3,186) patients diagnosing VTE during hospitalization in the authors’ service. Four of those 174 patients did not receive any VTE prophylaxis. Of those 170 patients who developed VTE during hospitalization and had VTE prophylaxis, 19 missed VTE prophylaxis for ≥3 days. Unadjusted risk factor analysis for VTE resulted in mSOFA score significance. However, after adjusted analysis for inflammation (CRP) and thrombosis (D-dimer), surrogate laboratory variables, were significant risk factors for VTE development, explaining part of the mSOFA significance.

Considering only the 170 patients who developed VTE during their stay in the service and received venous thromboembolism prophylaxis, the VTE proportion was 5.3%. Multiple systematic reviews and meta-analyses reported variable VTE incidence in patients with COVID-19 according to the diagnostic approach, whether passive upon presentation of clinical manifestation (9.5%) or active search (14.1%),^26^ and critical clinical condition, regardless of the patient being in intensive care (13.7%) or on ward beds (3.5%).^1^^,^^2^^,^^4^^,^^12^^,^^18^^-–^^21^ When in intensive care and under an active diagnostic search protocol, the incidence of VTE can increase up to 4 times, with a prevalence of 48% (95% Confidence Interval, 95% CI: 0.33 to 0.63).^2^ There is even a discussion about whether patients with COVID-19 develop more thrombosis compared to non-COVID-19 patients with the same severity.^12^ However, in those prevalence studies of VTE in COVID-19 patients, the use of thromboprophylaxis is not invariably mentioned. In patients without COVID-19, several studies that employed screening for DVT in critically ill patients showed that admission to ICU was a risk factor for VTE (RR: 1.8–2.9). The incidence of DVT in medical ICUs is very high, particularly in patients receiving no prophylaxis (25%–31%), compared with those that receive some form of prophylaxis (11%–16%).^18^ Interestingly, in the present data, the incidence was lower than these prior reports, reflecting better prophylaxis practice, although residual risk still exists, of which the clinician should be aware.

A distinct aspect considered in the present study was the VTE diagnosis only with objective confirmation, either by venous ultrasound of the limbs or by diagnostic pulmonary CT angiography. This contrasts with studies without confirmatory tests for VTE, which may overestimate the diagnosis of thromboembolic phenomena based on criteria such as the elevation of D-dimer,^27^ change in the clinical situation, for example, where it could have been made for some patients unable to do the VTE confirmation test.

According to the passive search methodology used in this analysis, there are some potential limitations to be discussed. The search for those patients was based on the strategy the authors employed to restrict the search for patients, which was the use of anticoagulants in doses above the prophylactic. Thus, the authors cannot rule out the possibility that some cases with VTE diagnosis could have been excluded in the first screen due to the patient's very low body weight or without treatment, although unlikely.

Regarding patients’ age, the present study is in accordance with the literature.^24^^,^^28^ The group of patients with VTE was older than the group without VTE. Since long ago, several epidemiological studies^29^ and guidelines^18^ have established that the incidence of VTE increases exponentially with age. In a study carried out in Oslo,^30^ the incidence of VTE increased from 1:10,000 at 20 years of age to 1:1,000 at 50 years of age. In a North American study in Worcester,^31^ authors found that from the fifth decade of life onwards, the risk of VTE practically doubled with each subsequent decade (RR = 1.9). However, it is still not known whether that is due to changes in coagulation mechanisms with aging or to the presence of thrombogenic comorbidities.

Regarding disease severity, assessed by both the mSOFA score and the presence of acute kidney injury, patients with confirmed VTE (G1) had a more severe clinical condition compared to patients without VTE (G2) in the present study. It resulted in longer hospital stay and higher mortality (Table 1). This allows us to postulate that, even in the face of a severe COVID-19 infection requiring hospitalization, the most severe would be the most likely to develop more thromboembolic phenomena. There is also the possibility that the VTE itself had worsened the patient's clinical condition. Indeed, publications^11^^,^^12^ are evidencing COVID-19 intensive care patients’ worse outcomes, including higher VTE rates, in comparison with intensive care patients without COVID-19 infection. Among our 245 VTE COVID-19 patients, 135 (55%) were admitted to intensive care and 110 (45%) to wards.

Mortality of our COVID-19 patients was high, reaching 29.3% (28.7% for non-VTE patients and 36.7% for VTE patients) (Table 1). This high mortality can be explained by the very nature of our service, which was a reference for patients with critical COVID-19, and probably also due to disparities in the Brazilian public health system.

A relevant finding among the 245 VTE patients was that 29 patients (12%) already had a diagnosis of VTE when they were admitted to the present service and for another 42 (17%) the diagnosis occurred at the time of admission to the present service. Except for those 71 patients, almost 30% of patients did not receive VTE prophylaxis in the present service before the event. On the other hand, for 50% of patients who developed VTE during hospitalization, this event occurred within the first week of hospitalization, reflecting the potential immunothrombotic impact related to COVID-19 infection.

Nevertheless, the authors believe that some of the 174 patients with VTE during hospitalization could have been prevented by adequate prophylaxis. For 4 patients, no prophylaxis was prescribed. Moreover, of the 170 patients who were prescribed prophylaxis, 53 patients (31%) did not receive all prescribed doses. Here, there are diverse plausible hypotheses, such as the patient had a bleeding event or, for example, the patient was unable or away from the bed ward to do some exam or test at another department at the time of the prophylactic drug administration, or other reasons not registered at the medical record. Of note, the VTE occurred with 19 patients (11%) who did not receive prophylaxis for 3 or more consecutive days. This fact raises an important topic: the failure to receive prophylaxis may be a risk factor for immunothrombosis. In fact, as early as 1999, Samama et al., in the MEDENOX VTE prophylaxis study,^32^ used these 3 days without prophylaxis as an exclusion criterion, resulting that those patients who were bedridden for 3 days or more were not allowed to participate in their study. This may be related to the fact that 3 days without prophylaxis is the limit to putting the patient at risk for immunothrombosis and VTE occurrence. In another study, of orthopedic surgical patients,^33^ also observed that the loss of prophylaxis doses led to a higher incidence of VTE, and this became significant from 2‒4 daily doses failure of enoxaparin administration. Although the authors agree with this idea, its confirmation requires more specific studies. When comparing the patients who had failures greater than or equal to 3 days in receiving prophylaxis (Table 4), the authors found that their disease severity was not different from those without discontinuity; however, their length of stay was 50% longer indicating poorer recovery.

Failures in pharmacological therapy vary widely in the literature. Errors can lead to delays in drug application or even loss of doses.^34^ These errors range from 3.5% to 22%, with dose losses reaching 19%. In the present study, the number of one-day failure reached 31%, with 11% not receiving 3 or more consecutive doses. However, it was observed that one of the reasons for not receiving the dose might have been due to significant bleeding, which occurred in 10% of these patients. Major bleeding events rarely occurred in both groups, but they deserve detailed studies in exceptionally large samples of patients using anticoagulants.

In this study, 107 patients received full prophylaxis and still developed VTE. Indeed, since the late 1990s, Samama et al.^32^ already showed that, even when receiving prophylaxis, 1% of patients developed clinical VTE (5% with VTE detected if there was an active search strategy). In the present study, the authors found a rate of 3.3% of patients who, despite receiving prophylaxis, developed VTE (107/3,246).

Factors associated with mortality in COVID-19 patients have been the subject of much discussion. Some authors studied factors associated with mortality and VTE and minimized their risk in black patients and the risk was increased in the elderly who are obese and/or have co-morbidities.^12^ Some medicines have also been associated with prognosis, such as the Angiotensin-Converting Enzyme Inhibitors (ACEI),^12^ statins, and platelets anti-aggregation drugs,^26^ decreasing the risk of VTE and/or mortality. The contrary was observed during the use of Chloroquine, with increased VTE risk and mortality.^12^ In the current study, the authors found an increased risk for thinner patients, an unexpected finding, maybe implying a larger degree of malnutrition related to COVID-19′s intense inflammatory status. Unfortunately, we do not have information on how long those patients were already sick before being referred and admitted to the present service.

In the present study, the authors found increased mortality of COVID-19 patients, along with a prolonged hospital stay. These findings are not concordant with other studies negating the importance of VTE on COVID-19 mortality and causing only a prolonged hospital stay.^35^ However, there is a lack of consensus regarding this subject, and studies with larger samples will be of importance.^36^

When the authors analyzed laboratory markers for thrombosis, a large temporal overlap of the thrombotic event and the highest level of D-dimer presented by the patient (Fig. 2) were seen. Both alterations occurred in the first week of hospitalization. In an earlier study, the authors showed that, in this same population, D-dimer values higher than 4,000 ng/mL had a significant relationship with mortality, although this mortality was not necessarily due to thrombotic events. This suggested that this analyte might also be showing the intensity of the systemic inflammatory process.

The authors also analyzed C-reactive protein, fibrinogen, and platelets in patients with thrombosis *versus* those without thrombotic phenomena. In the VTE group, all their greatest variations occurred in the first week of hospitalization. This suggests that in COVID-19, whose strains at the time of the study did not include the Omicron variant, the highest peak of inflammation occurred in the first week of hospitalization.

The present manuscript has some limitations. It was a retrospective, single-centre study and therefore results are related to the practice patterns at this center. Second, this analysis did not include patients’ follow-up after discharge, hence many patients were censored for survival analyses. Third, although there were some missing data, especially for laboratory variables, the authors performed analysis for complete cases, since we could not assume missing at random for imputation. Fourth, the initial VTE screen was triggered by a related indicator of VTE diagnosis (i.e., use of full dose anticoagulation), but this was confirmed by individual patients’ medical records review for their diagnosis and included results of venous Doppler ultrasound and chest angiotomography. Fifth, given the retrospective nature of the present study, the authors , unfortunately, do not have all data that would be considered relevant for regression analysis, so these results should be cautiously interpreted.

## Conclusions

The incidence of proven VTE in patients with severe COVID-19 in the present cohort was 7.7%, despite 87% full compliance with VTE prophylaxis. Those patients were more critical, had a higher mortality rate, worse SOFA score, higher incidence of acute renal failure, and, on average, a 50% longer hospital stay. The clinician must be aware of the diagnosis of VTE, even in patients receiving proper prophylaxis.

## Authors' contributions

CAL, FARG, BAMPB, and CAL did the literature search. FARG, BAMPB, AJRP, LAMF, EMT, NMS, AJSD and AL designed the study. CAL, FARG, BAMPB, and EMT collected the data. CAL, FARG, BAMPB, EMT and AL analyzed the data. CAL, FARG, BAMPB, APC, AJRP, SFP, CPG, LAMF, EMT, NMS, AJSD, CBM, and AL interpreted the data. CAL, FARG, BAMPB, APC, AJRP, SFP, CPG, LAMF, EMT, NMS, AJSD, CBM, and AL wrote and reviewed the paper.

## Conflicts of interest

The authors declare no conflicts of interest.
